# Use of implementation science methods to design *Wellness Hub*, a responsive program to address long-term care and retirement homes’ challenges during the COVID-19 pandemic

**DOI:** 10.1186/s12913-025-13997-8

**Published:** 2026-01-24

**Authors:** Christine Fahim, Keelia Quinn de Launay, Vanessa Bach, Jessica Firman, Claire R. Gapare, Vincenza Gruppuso, Ayaat T. Hassan, Ana Mrazovac, Temi Odunuga, Nimitha Paul, Lisa Strifler, Alyson Takaoka, Elikem Togo, Hui Juan Chelsea Gao, Jamie M. Boyd, Sharon E. Straus

**Affiliations:** 1 Knowledge Translation Program, Li Ka Shing Knowledge Institute, St. Michael’s Hospital, Unity Health Toronto, 209 Victoria Street, Toronto, ON M5B 1T8 Canada; 2 Institute of Health Policy, Management and Evaluation, University of Toronto, 155 College Street, Suite 425, Toronto, ON M5T 3M6 Canada; 3 30 Bond Street, Toronto, ON M5B 1W8 Canada

**Keywords:** Real-world problems, Implementation science, Implementation practice, Integrated knowledge translation, Long-term care, Implementation mapping, Theoretical frameworks, COVID-19

## Abstract

**Background:**

Long-term care and retirement homes (LTCH/RH) faced systemic challenges that were exacerbated by the COVID-19 pandemic. Homes faced three major challenges over the course of the pandemic: implementing infection prevention and control (IPAC) practices, facilitating COVID-19 vaccine uptake and confidence, and addressing staff well-being and burnout. This manuscript describes the use of implementation science methods to design an evidence-based, theoretically-rooted program titled the *Wellness Hub* to support LTCH and RH to navigate real-time COVID-19 challenges.

**Methods:**

Challenges facing homes were categorized to theoretical constructs using the Theoretical Domains Framework (TDF) and the Consolidated Framework for Implementation Research (CFIR). Implementation mapping was used to identify strategies to mitigate barriers and leverage facilitators at the individual level using the SELECT tool (rooted in Michie’s Behaviour Change Wheel) and at the organizational level using the CFIR-Expert Recommendations for Implementing Change (ERIC) matching tool. A multidisciplinary project team and steering committee reviewed the results and contextualized identified strategies to design the *Wellness Hub Program* components.

**Results:**

Twelve TDF domains and 18 CFIR constructs were identified as barriers and/or facilitators to implementation of IPAC protocols, COVID-19 vaccine uptake, and staff well-being programs. Via the SELECT tool, we identified 14 implementation strategies to target individual-level change. An additional four strategies for organizational and systems-level implementation change were identified via the CFIR-ERIC mapping tool. The resulting *Wellness Hub Program* included: town halls, implementation coaches, promotion for LTCH/RH wellness days, creation of infographics and educational resources (including an open-access resource repository), a weekly newsletter summarizing LTCH/RH directives, a vaccine champions program and e-learning course, modelled change, an IPAC-self-assessment tool, seed funding, vaccine incentives, access to off-site COVID-19 testing, monthly community of practice meetings and use of opinion leaders.

**Conclusion:**

Use of implementation science methods facilitated the design of a responsive support program to address LTCH and RH’s real-time, evolving COVID-19 challenges.

**Study registration:**

https://osf.io/hkfae.

**Supplementary Information:**

The online version contains supplementary material available at 10.1186/s12913-025-13997-8.

**Table:** 

Text box 1. Contributions to the literature
• Long-term care and retirement homes experienced evolving challenges during the COVID-19 pandemic; in particular, challenges related to infection prevention and control, vaccine uptake, and staff well-being were pervasive in the province of Ontario, Canada.
• Implementation science methods, including the use of a co-creation approach, theoretical frameworks and implementation mapping were used to develop the *Wellness Hub*, a responsive program that evolved with homes’ needs in real time.
• This study shows how implementation science methods can be used to address real-time challenges during health emergencies.

## Background

Long-term care homes (LTCHs) and retirement homes (RHs) have consistently faced systemic challenges related to staffing shortages, funding constraints, outdated infrastructure, and lack of standards and regulatory policies [1]. Wong et al. estimate that over $20 M (CAD) between 1998 and 2020 was dedicated to commissioning over 80 reports to describe challenges and recommendations for Canadian LTCH [2]. Despite these well-known challenges, systemic gaps in LTCH and RH remained unaddressed. It was in this context that the COVID-19 pandemic struck, creating an unparalleled crisis for Canadian LTCH/RH.

In 2020, COVID-19 deaths in Canadian LTCH/RH were significantly higher than any other nation, estimated at 81% compared to 31% in the United States and 27% in Australia [3]. The province of Ontario was especially affected, due to coordination gaps between homes, public health units and hospitals, more shared rooms, fewer care hours for residents, fewer resources and less oversight into care processes [4].

To respond to this challenge, we aimed to develop a support program to address LTCH/RH needs during and beyond the COVID-19 pandemic. Foundational work to inform program development included conducting needs assessment interviews with LTCH and RH leaders in Ontario [5]. Interviews were conducted to determine the challenges facing homes, and ensure our resulting program would meaningfully address homes’ needs. Data were collected iteratively between February 2021 and July 2022 which facilitated the identification of needs that changed over time, as the pandemic evolved. During this period, leaders identified challenges broadly relating to three categories: (1) challenges implementing infection prevention and control (IPAC) protocols and measures, (2) challenges facilitating COVID-19 vaccine access and uptake, and (3) staff well-being, burnout, and mental health challenges exacerbated by the pandemic [5].

To optimize implementation success, we used implementation science methods to develop a program that was more likely to be acceptable, effective, and sustainable in real-world conditions [6]. The use of theoretical frameworks provides a systematic approach to identify the determinants and mechanisms as to why an intervention may or may not be effective [6]. These frameworks can be used to categorize barriers and facilitators to implementation, providing insights on *where* change needs to occur (e.g., at the individual level, organizational level) and *why* actors may or may not change their behaviours [7]. Through a process called implementation mapping, theoretical frameworks can also be used to inform *how* programs can leverage facilitators while mitigating barriers [8, 9]. Programs that are designed without the use of theory may not address the mechanisms that drive change, resulting in misalignment and limited applicability. Martin Eccles, Emeritus Professor of Clinical Effectiveness, is famously quoted warning against implementing programs that “seemed like a good idea at the time” [10, 11]. Instead, the systematic use of theories and frameworks to implement evidence-based interventions should be used to improve reproducibility, scalability, efficiency, and relevance of resulting programs [7, 11].

The purpose of this manuscript is to describe the process of designing the *Wellness Hub Program* to address LTCH and RH’s COVID-19 challenges, using theoretical frameworks and implementation mapping.

The study objectives were as follows:

1. Categorize challenges related to the implementation of IPAC, vaccine, and staff well-being practices in LTCH/RH using theoretical frameworks to systematically identify barriers and facilitators to change.
2. Use implementation mapping to identify strategies that mitigate identified barriers and leverage identified strategies.
3. Contextualize identified strategies to the LTCH and RH context in partnership with multidisciplinary actors in order to design the *Wellness Hub Program* components.

## Methods

### Foundational work

Challenges facing home leaders were identified via semi-structured, key informant interviews conducted with 91 LTCH/RH leaders across 33 LTCH and 14 RH [5]. Participants were leaders in for-profit (63.8%) and non-profit (36.2%) homes located mainly in the Greater Toronto Area and Ottawa-Champlain Regions of Ontario, Canada. Detailed methods and interview findings are reported elsewhere [5]. Leaders identified ten challenges related to IPAC implementation (e.g., lack of resources to deliver IPAC education, resource shortages); six challenges related to COVID-19 vaccine access and uptake (e.g., mistrust around vaccine safety, lack of vaccine availability); and three challenges related to ability to address staff well-being concerns (e.g., lack of access to supports; fear of stigma). In addition, 13 themes pertaining to opportunities or strategies used to address these challenges were identified, although the prevalence and use of these strategies varied considerably by home [5].

### Mapping to theoretical frameworks

To inform *Wellness Hub* program development, identified challenges were mapped to theoretical frameworks to systematically identify barriers and facilitators that impact homes’ ability to implement evidence-based practices (e.g., IPAC protocols, see Appendix 1) to address each of the challenges. A rapid analysis approach was used to meet the demands of these urgently needed program supports. First, the research team familiarized themselves with the needs assessment data and developed a codebook by double-coding five needs assessment interviews [12]. This codebook was then used to double-code fifteen interviews (KQD, LS, ET, AH, JF); the remaining interviews were single-coded by two researchers (AH, LS). Following the indexing of the codes, the research team themed the data to describe challenges, supports and strategies related to infection prevention and control, vaccine uptake and confidence, and staff mental health and well-being. These themes are comprehensively described elsewhere [5].

We then mapped the resulting IPAC, vaccine, and staff well-being challenges to theoretical frameworks using a deductive coding approach, as outlined by Atkins et al. [7]. Each theme was coded as a barrier or facilitator construct using theoretical frameworks, specifically the Theoretical Domains Framework (TDF) or the Consolidated Framework for Implementation Research (CFIR) [13–15]. The TDF is a meta-framework of over 30 behavioural psychology theories describing barriers and facilitators to individual change. It outlines 14 domains that impact behaviour change, typically at the individual level. Domains include *knowledge; skills; social/professional role and identity; beliefs about capabilities; optimism; beliefs about consequences; reinforcement; intentions; goals; memory attention and decision processes; environmental context and resources; social influences; emotions; and behavioural regulation* [13]. The CFIR is a meta-framework that outlines five domains (*Intervention characteristics*,* Inner Setting*,* Outer Setting*,* Characteristics of Individuals*,* and Process*) that impact implementation [15]. Each domain includes various constructs; for instance *culture* and *leadership engagement* are constructs within the *Inner Setting* domain [15]. The TDF was used to categorize barriers and facilitators at the individual level and the CFIR, in particular the constructs of the *Inner Setting* (which represent the context at the individual LTCH or RH level) and the *Outer Setting* (which represent the broader context of the city, province and country) to categorize barriers and facilitators at the organizational or systems level [13, 15].

Three researchers (KQD, ET, LS) independently categorized the themes using the TDF and CFIR domains. Data were double-coded by at least two researchers (KQD, ET); conflicts or discrepancies were discussed and resolved using a consensus process. All categorized data were reviewed one final time by one researcher (KQD) to ensure consistency in coding and presentation of findings.

### Reflexivity statement

Our research team recognizes that positionality is intersecting and fluid, and that characteristics and viewpoints may impact processes of data collection and analysis. The research team included women of diverse roles, races/ethnicities, and ages. The team included individuals who work with older adults as clinicians, researchers, caregivers and those with loved ones in long-term and retirement home care. We followed the Knowledge Translation Program Intersectionality and Bias Reflection guides in efforts to minimize bias to data collection and analysis [16]. Research staff were not known to study participants.

### Implementation mapping

Implementation mapping is the process of identifying strategies that are theoretically-linked to the mechanisms that can impact behaviour change [9]. Theoretical frameworks can support the identification of appropriate strategies to mitigate identified barriers and leverage identified facilitators to behaviour change. For instance, the use of incentives to facilitate vaccine uptake would not be a suitable strategy for homes unable to access vaccines due to country-wide shortages. To identify strategies to mitigate identified barriers and leverage facilitators, researchers used the SELECT tool and the CFIR-ERIC matching tool [8, 17]. The SELECT tool provides an overview of Michie et al.’s guidance on how to integrate use of the TDF with the COM-B behaviour change wheel, a behaviour change theory that posits that behaviour can only change if the capability, opportunity, and motivation to do so exists. TDF domains align with the COM-B and can support identification of whether changes to an individual’s capability, opportunity and/or motivation need to take place in order to bring about the desired behaviour change. The COM-B behaviour change wheel then outlines nine categories known as intervention functions that can be used to bring about change related to capability, opportunity, and/or motivation [8, 9, 14]. For instance, the intervention function of “education” can be used to address challenges related to *knowledge* (identified via the TDF) that correspond to the Capability domain of behaviour. The SELECT tool further supports use of the COM-B by providing a list of implementation strategies, or specific techniques, that can be used to operationalize each intervention function. These strategies were derived from the Cochrane Effective Practice and Organization of Care framework and evidence reviews [18]. For example, implementation strategies corresponding to the “education” intervention function include “educational meetings or educational materials”. Using this process, the SELECT tool was used to identify implementation strategies to change individuals’ behaviour regarding IPAC practices, COVID-19 vaccine uptake, and staff well-being. A similar process was used to identify strategies to address barriers at the organizational level, identified using the CFIR. CFIR constructs were mapped to corresponding expert-endorsed strategies using the CFIR-ERIC mapping tool [15, 17]. The Expert Recommendations for Implementation Change (ERIC) is a list of 73 implementation strategies identified by implementation science experts using a Delphi method [17]. Experts ranked a list of strategies against the CFIR to identify the strategies that they believed best addressed each domain [17]. The CFIR-ERIC mapping tool is an Excel-based web tool that allows users to input the CFIR barriers relevant to their project. The tool then provides an output that shows the top expert-endorsed strategies for each of the barriers, alongside the percent endorsement of each strategy by experts (the higher the percent, the higher the level of endorsement by experts).

One researcher (KQD) conducted intervention mapping, matching the coded TDF and CFIR barriers to strategies using the SELECT and CFIR-ERIC tool, respectively. CFIR-ERIC strategies with endorsements greater than 50% were retained as potential options for implementation strategies [17]. Given the considerable overlap between the TDF and the CFIR, as well as the SELECT and ERIC strategies, duplicate strategies were removed by one researcher (KQD) and reviewed by an implementation scientist (CF). The final list of potential implementation strategies were retained as a list of options to inform design of the *Wellness Hub Program.*

### Design of the Wellness Hub Program

A core team of researchers at the Knowledge Translation Program, Unity Health Toronto, led this project. The core team included two implementation scientists (CF, SES) and research coordinators who were tasked with conducting the qualitative and mapping analysis. To ensure the *Wellness Hub Program* was co-developed with relevant LTCH and RH parties, the research team assembled a broader project team that included representation from national (e.g., Healthcare Excellence Canada), provincial (e.g., Public Health Ontario), and regional (e.g., Toronto Central Local Health Integrated Network) public health and government organizations; organizations that inform care for older adults (e.g., Regional Geriatric Programs); Long-Term Care and Retirement Home Associations (e.g., Ontario Long Term Care Association); family and caregiver organizations (e.g., Family Councils Ontario); professional organizations (e.g., Ontario Personal Support Worker Association); physicians; researchers; front-line LTCH/RH staff; and caregivers [19]. In addition, a steering committee of 20 individuals that were representative of the broader project team was formed.

Using an integrated knowledge translation approach, the steering committee was iteratively presented with the challenges facing the homes and proposed *Wellness Hub* components identified by the project team [20]. Over a 16 month period, the steering committee reviewed and reflected on the findings of the Wellness Hub data including the findings of the needs assessment interviews, evidence review (Appendix 1), barriers and facilitators mapping, and preliminary evaluations, including homes’ feedback on implemented strategies. Findings were shared with the steering committee via plain language e-mail summaries or virtual presentations. This iterative process allowed our team to adapt our strategies as home needs and the pandemic evolved. For instance, some of the earliest *Wellness Hub* components focused on strategies to address IPAC; however, as vaccines emerged, so did proposed strategies to facilitate vaccine uptake and confidence. At each touch point (e-mail or virtual meeting), committee members were asked to reflect on the findings, identify any gaps/missing components, and provide guidance on the components or strategies that could be used to operationalized identified theoretically-rooted strategies.

Once program components were identified by the steering committee, members of the broader study team were engaged to further operationalize the strategies. For example, education materials were one of the strategies identified via mapping to address knowledge gaps. Members of the research team compiled evidence-based educational materials, and where these did not exist, created original resources. The materials were then reviewed by content experts and LTCH/RH knowledge users within the broader study team prior to integration of the resources into the *Wellness Hub Program* offerings.

## Results

### Barriers and facilitators mapping to theoretical domains

Tables 1, 2 and 3 outline the mapping of barriers and facilitators relevant to IPAC, vaccine, and staff well-being challenges to TDF domains and CFIR constructs. At the individual level, barriers and facilitators related to all dimensions of behaviour change (capability, opportunity and motivation) were identified. At the contextual level, most barriers and facilitators related to the *Inner Setting* (i.e., pertaining to individual LTCH or RH), though some system-level barriers at the *Outer Setting* were also identified.

**Table 1:** Barriers and facilitators to IPAC uptake, coded to TDF and CFIR

Barrier	Description of Theme	TDF Domains (COM-B)	CFIR constructs (CFIR Domain)
Delivering IPAC education in the LTCH setting was time consuming, resource intensive	Educating staff on IPAC measures was very time consuming and resource intensive, as leadership had to adapt standard IPAC education to the context of the COVID-19 pandemic and outbreaks.	• Environmental Context and Resources (Opportunity) • Knowledge (Capability) • Memory, Attention and Decision Processes (Capability) • Behavioural Regulation (Capability)	• Compatibility (Inner Setting) • Available resources (access to knowledge and information) (Inner Setting)
Staff did not implement IPAC guidance consistently	Sites experienced inconsistent implementation of IPAC protocols from their staff, such as not always social distancing, not maintaining consistent hand hygiene, and irregularity with how staff wore PPE.	• Intentions (Motivation) • Behavioural Regulation (Capability)	
Residents with limited capacity were not able to follow IPAC protocols	Staff have experienced challenges with ensuring residents with dementia are following IPAC protocols. There were challenges around keeping PPE on residents with dementia, ensuring that they maintained hygiene (i.e., handwashing), and maintaining isolation of ill residents.	• Behavioural Regulation (Capability) • Beliefs about Capabilities (Motivation)	• Compatibility (Inner Setting)
Homes were challenged to keep up with rapidly evolving protocols and with communicating changes to families/caregivers and staff, which led to confusion, fear, anxiety and anger	Directions and protocols were constantly changing, making it difficult for staff to keep track of current protocols and practices. This was especially true for RH who did not always have tailored public health guidance available to them. Many sites had difficulty keeping up with the updates as a result, and experienced challenges with effectively communicating the ongoing changes in IPAC protocols to staff, residents, and caregivers/families.	• Knowledge (Capability) • Emotion (Motivation)	• Policies & Laws (Outer Setting) • Available Resources (Inner Setting)
Resources shortages, including PPE and access to COVID-19 rapid tests	Sites experienced shortages or poor access to PPE for staff members, making it difficult to follow IPAC protocols. This was particularly an issue during the first COVID-19 wave, where there were shortages on all PPE, and was also seen later in the pandemic during a shortage of N95 masks following guidance changes. Following recommendations to employ rapid COVID-19 testing during the Omicron wave, sites experienced challenges in acquiring sufficient rapid COVID-19 testing kits in a timely manner.	• Environmental context and resources (Opportunity)	• Available resources (materials & equipment) (Inner Setting)
Lack of funding to cover costs related to N95 mask fit testing, staff training for IPAC protocols	While some homes had enough funding or resources in-house to support IPAC (e.g., N95 mask fitting; IPAC protocol training), other homes did not have the necessary resources in house nor sufficient funding to do so.	• Environmental context and resources (Opportunity)	• Available resources (funding) (Inner Setting)
Physical environment of homes not conducive to IPAC implementation	Homes experienced challenges around finding physical space for IPAC supplies, disinfecting large areas, and ensuring physical distancing in an environment that was not built to handle such expectations (e.g., lack of space, small physical space).	• Environmental context and resources (Opportunity)	• Structural characteristics (physical infrastructure) (Inner Setting) • Available resources (space) (Inner Setting)
PPE fatigue	Staff members and caregivers became tired of wearing PPE, due to discomfort that comes from wearing many layers of PPE consistently throughout their shift; this worsened in warmer weather. This led to PPE burnout and decreased compliance to PPE in many instances.	• Emotion (Motivation)	
Family pushback on IPAC protocols	The pandemic led to new levels of distrust and pushback from caregivers/family members to loosen restrictions. Family members called to complain about not being allowed to visit. Some family members requested residents only be cared for by vaccinated staff members, creating tensions between staff.	• Beliefs about Consequences (Motivation) • Social professional role/identity (Motivation) • Social influences (Motivation) • Emotion (Motivation)	
Fears of returning to normal and loosening IPAC restrictions	Some found that adapting to the earlier stages of the pandemic easier because the focus was on constant change. Later in the pandemic, some individuals found it difficult to loosen IPAC restrictions, for fear of another outbreak. Some expressed a fear of “returning to normal”.	• Beliefs about Consequences (Motivation) • Emotion (Motivation)	
**Facilitator**	**Description of Theme**		
Having a dedicated IPAC manager/nurse/champion	IPAC representatives helped provide advice, answer questions, and supported each site and their staff to ensure IPAC protocols were properly followed.	• Behavioural regulation (Opportunity) • Social influences (Motivation)	• Available resources (access to knowledge & information) (Inner Setting)
Consistent communication with public health units	Some sites had regular communication with their local public health units that provided them with support, education, and information.	• Environmental context and resources (Opportunity)	• Partnerships & Connections (Outer Setting)
External supports from hospitals or public health unit (IPAC guidance, physical or financial resources)	Sites received a range of supports/ resources from local public health agencies and external organizations/ hospitals. There was also financial support (e.g., from provincial government) to homes to purchase PPE and cleaning supplies, and hire emergency services to provide onsite support.	• Environmental context and resources (Opportunity)	• Available resources (funding) (Inner Setting) • Structural characteristics (work infrastructure) (Inner Setting) • Partnerships & Connections (Outer Setting)
Use of multi-pronged strategies to disseminate IPAC updates to staff	Sites utilized a variety of strategies to disseminate IPAC information and updates to their staff, tailoring formats or leveraging communication strategies they already had in place. Strategies included huddles, town halls, emails, calls, handouts, bulletins, in combination with innovative, interactive, and informal approaches to sharing information.	• Memory, Attention and Decision Processes (Capability) • Behavioural Regulation (Capability) • Social Influences (Motivation)	• Communications (Inner Setting) • Available resources (access to knowledge & information) (Inner Setting)
Monitoring and audits on IPAC compliance	Sites monitored and conducted audits to ensure staff, caregivers, and visitors complied with IPAC protocols.	• Behavioural regulation (Capability) • Reinforcement (Motivation)	• External pressure (performance-measurement pressure) (Outer Setting)
Having leaders who are committed to transparency with staff, families/caregivers	Commitment to communication from leadership, and transparent, ongoing communication among staff, and between staff and caregivers/ families was a facilitator; this included open dialogue on challenges around adherence to IPAC guidelines and was perceived to foster trust.	• Environmental context and resources (Opportunity) • Social, professional role and identity (Motivation) • Emotion (Motivation)	• Available resources (access to knowledge & information) (Inner Setting) • Relational connections (Inner Setting) • Culture (recipient centeredness) (Inner Setting)
Homes with physical space conducive to IPAC measure implementation	Having a larger sized home facilitated following IPAC protocols on physical distancing and social isolation. Sites also leveraged other techniques, including restricting movement through cohorting.	• Environmental context and resources (Opportunity) • Behavioural regulation (Capability)	• Structural characteristics (physical infrastructure) (Inner Setting) • Available resources (space) (Inner Setting)
Having leaders with experience navigating public health emergencies	Sites with staff who either worked through previous pandemics, or earlier COVID-19 waves, were able to help sites navigate the pandemic and associated outbreaks.	• Knowledge (Capability) • Skills (Capability) • Social/professional role and identity (Motivation) • Beliefs about consequences (Motivation)	• Available resources (access to knowledge & information) (Inner Setting)

**Table 2:** Barriers and facilitators to vaccine uptake, coded to TDF and CFIR

Barrier	Description of Theme	TDF Domain (COM-B)	CFIR Construct (CFIR Domain)
Lack of vaccine availability	Homes experienced challenges with COVID-19 vaccine availability due to interim supply chain issues (i.e., Canada-wide 2nd dose delays) – this delayed vaccination of their staff.	• Environmental context and resources (Opportunity)	• Critical incidents (Outer Setting) • Available resources (materials & equipment) (Inner setting)
Mistrust around vaccine safety	There was mistrust from staff, caregivers, and residents around vaccine safety due to the following reasons: - Rapid vaccine approval timelines - Changes in vaccine schedule - Fears about long-term effects and vaccine safety and particularly around fertility, breastfeeding, and pregnancy Members of marginalized communities also experienced mistrust rooted in systemic and historical mistreatment by government and health institutions.	• Beliefs about consequences (Motivation) • Emotion (Motivation) • Knowledge (Capability) • Environmental context and resources (Opportunity)	• Local attitudes (Outer Setting) • Available resources (access to knowledge & information) (Inner Setting) • Innovation evidence-base (Innovation Domain; i.e., perceptions of COVID-19 vaccines)
Belief that the vaccine, particularly boosters, will not improve or impact health outcomes	Some staff did not believe the vaccines would improve health outcomes; this sentiment was further driven by positive COVID-19 cases and continued COVID-19 restrictions after vaccination.	• Beliefs about consequences (Motivation) • Optimism (Motivation)	• Tension for change (Inner Setting)
Lack of knowledge, access or ability to get to a vaccine clinic	Staff experienced challenges with accessing vaccine clinics, some of which were due to the following: - Lack of transportation to the vaccine clinic - Inability to find local vaccine clinics - No protected time to travel to a vaccine clinic (staff would rather wait until the vaccines were available at their site) - Inability to book an appointment following the new online vaccine clinic booking system; compounded with long waitlist times	• Environmental context and resources (Opportunity)	• Available resources (materials & equipment) (Inner Setting)
Families’ concerns about vaccine safety impacted vaccine uptake among residents who do not have capacity to provide consent	Families who did not want to get vaccinated prevented their loved ones (residents) from receiving the vaccines.	• Social influences (Opportunity) • Intentions (Motivation)	
Beliefs that vaccine mandates are an infringement on labour laws, personal liberties	Sites experienced staff pushback against vaccine mandates, voicing their concerns that it was against their rights and that they were being forced by their organization to get the vaccine.	• Emotion (Motivation) • Reinforcement (Motivation)	• Policies & Laws (Outer Setting) • Incentive systems (Inner Setting)
**Facilitator**	**Description of Theme**		
Individual-level strategies to promote vaccine confidence	Sites had success with targeting staff members at an individual-level and engaging leadership to educate staff on the vaccine, promote uptake, and provide access to evidence-based information.	• Social, professional role and identity (Motivation) • Knowledge (Capability) • Beliefs about consequences (Motivation) • Social influences (Opportunity) • Reinforcement (Motivation)	• Communications (Inner Setting) • Available resources (access to knowledge & information) (Inner Setting) • Relational connections (Inner Setting) • Culture (recipient centeredness) (Inner Setting) • Incentive systems (Inner Setting)
Organizational level strategies to promote vaccine confidence	Sites ensured that staff were provided with evidence-based information and education about the vaccine to combat misinformation, including information to explain the rapid approval process. Sites found that informational town halls were an effective strategy for increasing vaccine uptake. Sites utilized opinion leaders and vaccine champions to promote vaccine uptake. Strategies ranged from bringing in staff to speak to historically marginalized populations, having management being the first to get vaccinated, holding vaccine campaigns with champions, and bringing in team members who were negatively impacted by COVID-19 to speak about their experiences.	• Environmental context and resources (Opportunity) • Social, professional role and identity (Motivation)	• Compatibility (Inner Setting) • Culture (recipient centeredness) (Inner Setting) • Partnerships & connections (Outer Setting)
Incentives to support vaccine uptake	Some sites provided resources to staff to be vaccinated. This included paying transportation and parking fees for staff as an incentive to vaccination.	• Reinforcement (Motivation)	• Incentive systems (Inner Setting)
Improving ease of access to vaccinations for LTCH/RH populations	Sites’ connection to IPAC supports including IPAC hubs facilitated access to vaccine supply and appointments. Sites were also able to overcome logistical barriers to vaccine access by supporting staff with appointment booking or by leveraging mobile clinics/on-site clinics; this also included offering vaccines to caregivers while they were onsite visiting residents to facilitate/ encourage uptake.	• Environmental context and resources (Opportunity)	

**Table 3:** Barriers and facilitators to uptake of staff well-being strategies, coded to TDF and CFIR

Theme	Description of Theme	TDF Domain	CFIR Construct
Staff generally reported a lack of access to appropriate mental health and well-being supports in homes	Many homes did not have appropriate mental health supports available. As the pandemic progressed, some supports that were initially put in place were discontinued due to limited resources.	• Environmental context and resources (Opportunity)	• Available resources (knowledge & information; materials & equipment) (Inner Setting) • Structural characteristics (work infrastructure) (Inner Setting)
Leaders don’t know what to offer	Leaders were not aware of which resources or supports that could be offered to staff to address well-being and mental health challenges related to the COVID-19 pandemic and its effects; some leaders did not feel that they had the capacity or skills to offer these supports or that they should be the ones providing the supports.	• Knowledge (Capability) • Cognitive skills (Capability) • Social/professional role and identity (Motivation) • Environmental context and resources (Opportunity)	
When available, staff did not access Employee Assistance Programs and other well-being resources; stigma was perceived as a factor.	In situations where well-being resources and supports existed, there remained a lack of uptake. Staff did not have capacity to engage with them, and/or had concerns about stigma and privacy.	• Beliefs about consequences (Motivation) • Social influences (Opportunity)	
**Facilitator**	**Description of Theme**		
Home leaders willing and able to implement well-being strategies	To support staff and resident wellness, some sites implemented diverse strategies, which included: - Providing behavioural supports for residents through Behavioural Supports Ontario (BSO) - Limiting number of staff shifts and encouraging use of vacation time - Leveraging support from external organizations to promote staff wellness (e.g., wellness resources from Local Health Integration Networks [LHIN] and regional hospital networks; drop in counselors or psychogeriatric nurses) - Having clinical staff on site with expertise in promoting wellness (e.g., social worker, occupational health) - Offering Employee Assistance Programs - Hosting social activities and staff appreciation events, providing gift certificates - Distributing well-being resources (e.g., bulletins, self-serve resource table)	• Environmental context and resources (Opportunity) • Social influences (Opportunity) • Beliefs about consequences (Motivation) • Social, professional role and identity (Motivation)	• Culture (recipient centeredness) (Inner Setting) • Communications (Inner Setting) • Structural characteristics (work infrastructure) (Inner Setting) • Partnerships & Connections (Outer Setting) • Relational connections (Inner Setting)

A total of 12 TDF domains representing factors that impacted individual-level behaviour were identified. An additional 18 CFIR constructs representing organizational and system-level barriers and facilitators were identified. Notably, many CFIR constructs relating to the *Outer Setting* were only identified as facilitators, highlighting the importance of external supports available to homes during the pandemic (Tables 1, 2 and 3).

### Identification of implementation strategies to address barriers and leverage facilitators

Via the SELECT tool, TDF barriers and facilitators were mapped to the COM-B Behaviour Change Wheel Intervention Functions (Table 4) [8, 13]. The following intervention functions were identified as potential interventions to mitigate barriers and leverage facilitators: *Modelling* (addresses 7 domains); *Education and Enablement* (6 domains); *Persuasion and Training* (5 domains); *Environmental restructuring* (4 domains); *Coercion and Incentivisation* (3 domains), and *Restriction* (2 domains).

**Table 4:**
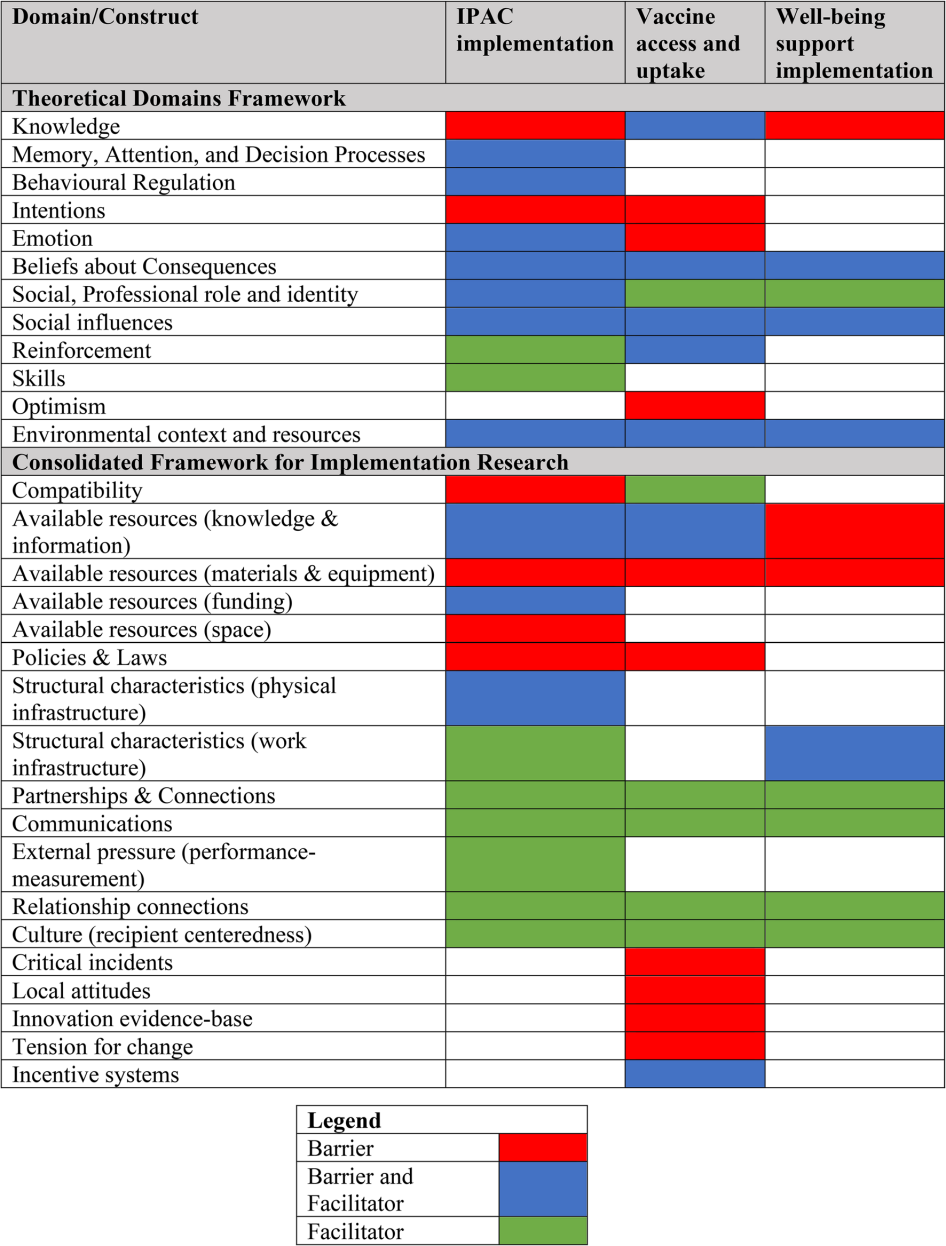
Mapping of implementation barriers to the Theoretical Domains Framework and the Consolidated Framework for Implementation Research

Using the SELECT tool, the following implementation strategies corresponding to these intervention functions were identified: conduct educational meetings, distribute educational materials, use champions, develop a community of practice, prepare LTCH leadership and staff to be active participants, secure public funding and contracting, alter payments to health workers, change service sites, offer incentives, model and simulate change, identify and use opinion leaders, use mass media, conduct local consensus discussions, and conduct educational outreach visits (Table 4).

The CFIR-ERIC mapping tool revealed four strategies with an endorsement percentage greater than 50% for the identified CFIR domains. These included: conducting educational meetings, developing educational materials, distributing educational materials and accessing new funding. These strategies overlapped with strategies identified via the SELECT tool, suggesting that these strategies can facilitate change across both the individual and organizational levels.

### Development of the Wellness Hub support strategy

Table 5 outlines the *Wellness Hub* components, their correspondence to the TDF domains and CFIR constructs, and identified implementation strategies.

**Table 5:** Wellness hub program -Implementation supports

Wellness Hub Implementation Strategy	Description	Implementation Strategies	Intervention Function / CFIR-ERIC Strategy
Use of a co-development, integrated KT approach in intervention design, and implementation	Engaged with homes to identify required supports; provided tailored supports to homes as required; continued collection of ongoing formal and informal stakeholder feedback to iteratively tailor and provide support strategies	• Conduct local consensus processes	• Persuasion
Town Halls	Town Halls with experts on vaccine information to build vaccine confidence, and on mental health and well-being in the LTCH/RH setting	• Educational meetings	• Education • Conduct educational meetings
Implementation coach	1:1 check-ins with an implementation coach to identify evolving barriers and facilitators and suggest corresponding theoretically-linked strategies	• Use of a learning collaborative • Conduct educational outreach visits	• Enablement • Training • Develop, distribute educational materials
Promotion for LTCH/RH Wellness days	Advertisement and encouragement to use CARE+, Wellness Day resources and a Peer-to-Peer Support Guide to support staff mental health and well-being	• Use mass media	• Persuasion
Infographics and online/printed resources	Infographics, resource booklets tailored to LTCH/RH on IPAC, Vaccine, and Wellness Days. Includes tailored educational materials upon home request (e.g., custom IPAC card for lanyard)	• Educational materials	• Education • Develop, distribute educational materials
Online resource repository	Resource repository with dedicated pages to IPAC implementation, vaccine access uptake and confidence, and well-being and mental health supports including original and existing resources	• Educational materials	• Education • Develop, distribute educational materials
Wellness Hub Newsletter	Weekly newsletter providing guidance on IPAC directives and updates (including evolving public health guidance), vaccine access (e.g., how to sign up for vaccine), vaccine confidence (e.g., FAQ about COVID-19 vaccines), advertisements for events and resources, opportunities to participate in a community of practice, lessons learned/spotlight on featured homes	• Educational materials • Community of practice	• Education
Vaccine Champions Program and e-Learning course	A self-directed vaccine champions e-course that provided homes with an opportunity to train staff to become peer vaccine champions	• Use of champions • Prepare leadership/staff to be active participants • Use train-the-trainer strategies	• Enablement • Persuasion • Training
Coaching and Modelling	Worked with LTCH/RH leaders to act as IPAC, vaccine and wellness champions in their own settings (e.g., how to plan an onsite wellness day for staff)	• Prepare leadership/staff to be active participants	• Enablement • Persuasion
IPAC self-assessment tool	Self-directed tool for homes to assess their IPAC implementation that identified gaps and corresponding resources to address gaps	• Preparing leadership/staff to be active participants • Educational materials	• Education • Enablement
$10,000 seed funding and vaccine incentives	Partnered with Healthcare Excellence Canada via their LTC + plus program to facilitate distribution of $10,000 (CAD) for quality improvement/pandemic navigation resources for LTCH/RH Homes had the authority to select how their funds would be used (e.g., wellness room, staff appreciation incentives, IPAC materials) Partnered with the “This is Our Shot” campaign to provide homes with vaccine incentives	• Public funding and contracting • Alter incentive/allowance structure	• Enablement • Incentivisation • Access new funding
Access to off-site COVID-19 testing	Provided access to at-home COVID-19 saliva testing and at-home or at work DBS COVID-19 dried blood spot antibody testing (the latter as part of a research project)	• Change service sites	• Environmental restructuring
Monthly community of practice meetings	Encouraged LTCH/RH with successes in a specific domain to share their stories and lessons learned; discussed new challenges and needs; provided featured resources and guest speakers	• Model and simulate change	• Modelling • Educational meetings
Opinion leaders	Via the implementation program and community of practice, we identified opinion leaders and supported LTCH/RH leaders to identify opinion leaders to support behavior change	• Identify and use local opinion leaders	• Persuasion
Vaccine mandates (external to Wellness Hub program)	Under the Ontario government directive, all LTCH/RH staff were mandated to receive at least 2 doses and 1 booster of the COVID-19 vaccine	• Mandate change	• Persuasion
Work with educational institutions (external to Wellness Hub program)	Many homes had access to Ontario’s hub-and-spoke model which partnered LTCH with academic and community hospitals to support IPAC implementation and uptake. Where possible, we aimed to facilitate these connections	• Work with educational institutions	• Training

*Wellness Hub* included 13 components: town halls, implementation coaches, promotion for LTCH/RH wellness days, infographics and online/printed educational resources, an open-access resource repository, a weekly *Wellness Hub* newsletter that summarized LTCH/RH directives (e.g. IPAC policies), a vaccine champions program and e-learning course, coaching for staff leadership on how to model change, an IPAC self-assessment tool, access to $10,000 in seed funding to put towards pandemic-related needs (through a partnership with Healthcare Excellence Canada), vaccine incentives, access to off-site COVID-19 testing, monthly community of practice meetings, and use of opinion leaders [21, 22]. Throughout, an integrated, knowledge translation approach was used to support program design as the pandemic evolved. The program components also allowed for tailoring based on the needs of each home (e.g., an implementation coach could recommend specific educational materials to address knowledge gaps in that home). Finally, two strategies external to the *Wellness Hub* offerings were leveraged to support change. The first was the COVID-19 vaccine mandates imposed by the Ontario government, which required all LTCH/RH staff to receive two doses and one booster dose of a COVID-19 vaccine, and subsequently facilitated vaccine uptake. The second was access to the Ontario LTC hub-and-spoke model, which partnered LTCHs with hospitals to facilitate case control management and IPAC implementation.

## Discussion

The COVID-19 pandemic placed unprecedented stress on the LTCH and RH sector which was already facing significant and longstanding challenges [23]. Our research team used an integrated knowledge translation approach and leveraged implementation science methods to develop the multipronged *Wellness Hub Program* to meet LTCH and RH’s evolving needs in real time. A myriad of barriers were identified, which impacted individuals’ and homes’ ability to implement interventions related to infection prevention and control, COVID-19 vaccine uptake, and staff well-being. A number of facilitators were also identified, including opportunities at the individual home and system level, to address many of these barriers. The resulting *Wellness Hub Program* included 15 components to address homes’ COVID-19 challenges.

COVID-19 placed a spotlight on LTCH/RH, yet there are few reported examples of programs that used implementation science to design evidence-based, theoretically-rooted supports to address home challenges [24]. Moreover, many initiatives that were implemented in long-term care during COVID-19 were focused on IPAC [25]. A systematic review showed that of 137 studies across 22 countries focusing on interventions in LTCH during COVID-19, half were focused on IPAC. The *Wellness Hub Program* is a unique example demonstrating the use of integrated knowledge translation and implementation science methods in real-time to iteratively respond to home needs as they evolved. Through this approach, we determined that IPAC was not the only major challenge, and that homes required support with COVID-19 vaccines, and critically, addressing staff well-being and burnout concerns. The *Wellness Hub Program* was implemented in 72 Ontario homes over a 2 year period and will be evaluated via a non-randomized trial to assess its impact on addressing homes’ needs [26].

*Wellness Hub’s* development aligns with expert calls to use implementation science methods to address ‘real-world, real-time’ problems during the COVID-19 pandemic. Experts argue that rapid and novel implementation methods were needed to address urgent pandemic-related problems, yet these were underused [27]. One challenge attributed to the urgency and rapid evolution of the pandemic was the lack of time and opportunity to design and conduct controlled, large-scale implementation science studies, particularly in hard-hit sectors such as long term care. Recognizing such challenges, our research team chose a pragmatic and iterative approach that prioritized the use of implementation methods to facilitate practice change.

We rapidly developed *Wellness Hub* by leveraging established, rapid analysis approaches and implementation strategy mapping tools such as the SELECT tool [8, 12]. This allowed us to efficiently identify homes’ COVID-19 challenges, categorize these challenges using theoretical domains, and match them to strategies, in real-time. This process facilitated a thoughtful, evidence-based and theory-informed selection of strategies that moved beyond traditional practices such as education and dissemination. We also observed heterogeneity between LTCH and RH, which necessitated tailored approaches to intervention delivery. As a result, our team developed *Wellness Hub* as a ‘menu’ of support programs and strategies from which homes could select. Identifying evolving needs and offering tailored strategies required deep, responsive engagement with homes, flexibility by our team, and adequate resourcing. Our pre-established relationships with LTCH and RH actors allowed us to rapidly co-develop and deliver the *Wellness Hub* program components as needs emerged. In some cases, our team also acted as a liaison between siloed groups with shared goals to support LTCH/RH (e.g. hospitals, health care organizations, LTCH/RH organizations). Our experience demonstrates the feasibility and criticality of engaging with knowledge users in health emergencies to design relevant and responsive programs that reduce resource waste. Our experiences add to the literature on how to effectively advance implementation science while conducting real-world implementation practice, in partnership with knowledge users [28]. Our experience also highlights the need to further advance implementation science methods for rapid response including opportunities to identify efficiencies to expedite data analysis, theoretical mapping, and evidence synthesis, which can be used beyond pandemics.

This study has limitations. *Wellness Hub* was informed via needs assessment data from homes in the Ontario region, many of which were in the Greater Toronto Area, and thus may not reflect the needs and experiences of LTCH and RH in other regions [5]. While *Wellness Hub* was co-developed with diverse parties, including resident organizations, caregivers, and LTCH and RH organizations, the steering committee and broader study team did not include representation from LTCH and RH residents. Finally, further research is needed to assess homes’ willingness and ability to use the *Wellness Hub Program* offerings and to evaluate the impact of the program on homes’ ability to address challenges and respond to the pandemic; this evaluation is currently underway.

## Conclusion

This manuscript describes the use of implementation science methods to develop a multipronged program called *Wellness Hub* to address long-term care and retirement home challenges during the COVID-19 pandemic.

## Supplementary Information

Below is the link to the electronic supplementary material.

Supplementary Material 1

## Data Availability

No datasets were generated or analysed during the current study.

## Abbreviations

CFIR: Consolidated Framework for Implementation Research
COM-B: Capability-Opportunity-Motivation Behaviour Change Wheel
ERIC: Expert Recommendations for Implementing Change
IPAC: Infection prevention and control
KTA: Knowledge to Action Cycle
LTCH/RH: Long-Term Care Homes/ Retirement Homes
TDF: Theoretical Domains Framework
