# Supplementary material for: Use of implementation science methods to design Wellness Hub, a responsive program to address long-term care and retirement homes’ challenges during the COVID-19 pandemic
Source: BMC Health Serv Res. 2026 Jan 24;26:264. doi: 10.1186/s12913-025-13997-8 (PMC12910846; doi:10.1186/s12913-025-13997-8)
Supplement: Supplementary file 1 — Supplementary Material 1 [file 12913_2025_13997_MOESM1_ESM.docx]

**Appendix 1 – Preliminary Work**

***Preliminary Work***

We formed a study steering committee and multidisciplinary project team to identify evidence and knowledge tools available to address each of the challenges related to IPAC implementation, vaccine access and confidence, and staff well-being and mental health challenges. Where possible, we aimed to ensure that the strategies we developed align with evidence and recommendations endorsed by public health decision makers in Ontario and Canada (e.g., Public Health Ontario).

*IPAC interventions*: We utilized the Public Health Ontario COVID-19 IPAC checklist for long-term care and retirement homes to guide implementation of IPAC practices across the homes (1). The checklist outlines 18 domains related to evidence-based IPAC measures, which were developed following a review of public health outbreak control measures and guidance, including a rapid review on COVID-19 transmission prevention in long-term care conducted by members of our team (1,2). To guide implementation of this checklist, we developed an online, IPAC self-assessment tool and an open-access IPAC implementation resource repository (3). In addition, we provided homes in the Greater Toronto Area with access to polymerase chain reaction (PCR) saliva testing, which provided rapid results on COVID-19 diagnosis and were accessible to symptomatic or high-risk exposure LTCH/RH staff, their household members, or essential care partners.

*Vaccine access and uptake*: We utilized the National Advisory Committee on Immunization (NACI) recommendations for COVID-19 vaccines (4). Given the evolving nature of these recommendations and fluctuating availability of access of the vaccines, we developed an online portal that provided information on the latest provincial (Ontario) and federal (Canadian) guidance regarding COVID-19 vaccines that provided homes with up-to-date information on the recommendations, eligibility, and availability of vaccines.

*Staff well-being and mental health challenges:* We identified a number of resources related to resident well-being during the pandemic, but few resources aimed at supporting LTCH/RH staff. Thus, we conducted an environmental scan of public health and mental health organizations in Canada (e.g. Public Health Ontario (5), Canadian Association for Mental Health [CMHA](6)) to identify resources related to mental health and well-being challenges identified via the needs assessment interviews. In particular, we aimed to identify resources related to the impact of COVID-19 on mental health (including post-traumatic stress disorder, grief and bereavement, anxiety and depression as secondary illnesses of burnout) and well-being (including burnout, moral injury, inability to practice mindfulness, and low staff morale/culture). A total of 247 resources were identified in this scan. For each of the resources, we outlined the source and the target audience (e.g., staff, caregivers). A staff member then reviewed the resources to ensure that they were rooted in evidence-based findings (e.g., randomized trial, systematic review). The resources were compiled into a well-being and mental health support package titled CARE+ (7). The package included resources and contact information to mental health supports and clinicians and crisis lines, by geographical area (7). The package was also translated to French to enhance accessibility in the province. Needs assessment interviews also showed that many staff were experiencing well-being challenges due to restrictions stemming from the COVID-19 pandemic (e.g., inability to work from home, loss of childcare)(8). To address this challenge, we developed a ‘wraparound resource package’, which included resources for self-isolation following COVID-19 exposure or diagnosis, resources for COVID-19 testing, food and grocery delivery, financial supports, and regional childcare supports (9). In addition, we developed a Peer-to-Peer support guide (10). To develop this guide, we conducted a Pubmed search to identify systematic reviews on peer-to-peer and workplace well-being interventions. For each identified review, we performed an AMSTAR2 critical appraisal assessment to appraise the quality of the evidence (11). Reviews with moderate to strong quality assessment were included in a peer support toolkit. Finally, a guide for planning a Wellness Day in congregate settings was developed in consultation with LTCH and RH stakeholders (12). The guide included a step-by-step outline of logistics that leaders should consider when developing and evaluating a Wellness Day to support staff morale (e.g., planning the wellness day, examples of activities, evaluation survey, activity sign-up sheets).

1. IPAC checklist for Long-Term Care and Retirement Homes. Public Health Ontario. September 2023. Available at: <https://www.publichealthontario.ca/-/media/Documents/I/2023/ipac-checklist-ltcrh.pdf>. Accessed June 13, 2024
2. Rios P, Radhakrishnan A, Williams C, et al. Preventing the transmission of COVID-19 and other coronaviruses in older adults aged 60 years and above living in long-term care: a rapid review. *Syst Rev.* 2020; 9(1):218

IPAC+. Wellness Hub. Available at: <https://wellness-hub.ca/ipac/>. Accessed June 13, 2024

1. National Advisory Committee on Immunization (NACI): Statements and Publications – COVID-19. Government of Canada. Available at: <https://www.canada.ca/en/public-health/services/immunization/national-advisory-committee-on-immunization-naci.html>. Accessed June 13, 2024
2. Mental Health. Public Health Ontario. Available at: <https://www.publichealthontario.ca/en/Health-Topics/Health-Promotion/Mental-Health> Accessed June 13, 2024
3. Mental Health Resources. Canadian Mental Health Association. Available at: <https://cmha.ca/find-info/mental-health/general-info/> Accessed: June 13, 2024
4. CARE+. Wellness Hub. Available at: <https://wellness-hub.ca/care/>. Accessed June 13, 2024
5. Fahim C, Hassan AT, Quinn De Launay K, Takaoka A, Togo E, Strifler L, et al. Challenges facing Canadian Long-Term Care Homes and Retirement Homes during the COVID-19 pandemic [Preprint: https://www.medrxiv.org/content/10.1101/2024.06.19.24308949v1]. 2024.

Wraparound Resources Package. Wellness Hub. Available at: <https://wellness-hub.ca/wraparound-resources/>. Accessed June 13, 2024

1. Peer Support Toolkit. Wellness Hub. Available at: <https://wellness-hub.ca/res/the-wellness-hubs-peer-support-toolkit/> Accessed June 13, 2024
2. Shea BJ, Reeves BC, Welles G et al. AMSTAR 2: a critical appraisal tool for systematic reviews that include randomised or non-randomised studies of healthcare interventions, or both. *BMJ* 2017;358;j4008
3. Wellness Day Planning Guide. Wellness Hub. Available at: <https://wellness-hub.ca/res/wellness-planning-day-guide/>. Accessed June 13, 2024
